# Oncolytic virus treatment differentially affects the CD56^dim^ and CD56^bright^ NK cell subsets in vivo and regulates a spectrum of human NK cell activity

**DOI:** 10.1111/imm.13453

**Published:** 2022-03-09

**Authors:** Michelle Wantoch, Erica B. Wilson, Alastair P. Droop, Sarah L. Phillips, Matt Coffey, Yasser M. El‐Sherbiny, Tim D. Holmes, Alan A. Melcher, Laura F. Wetherill, Graham P. Cook

**Affiliations:** ^1^ Leeds Institute of Medical Research, School of Medicine, University of Leeds Leeds UK; ^2^ Oncolytics Biotech Inc Calgary Alberta Canada; ^3^ Present address: Wellcome‐MRC Cambridge Stem Cell Institute University of Cambridge Cambridge UK; ^4^ Present address: Wellcome Trust Sanger Institute Cambridge UK; ^5^ Present address: School of Science and Technology Nottingham Trent University Nottingham UK; ^6^ Present address: Clinical Pathology Department Faculty of Medicine Mansoura University Mansoura Egypt; ^7^ Present address: Department of Clinical Science University of Bergen Bergen Norway; ^8^ Present address: Institute of Cancer Research London UK

**Keywords:** cell cycle, immunotherapy, lymphocyte trafficking, natural killer cells, NK cells, oncolytic virus, proliferation, reovirus

## Abstract

Natural killer (NK) cells protect against intracellular infection and cancer. These properties are exploited in oncolytic virus (OV) therapy, where antiviral responses enhance anti‐tumour immunity. We have analysed the mechanism by which reovirus, an oncolytic dsRNA virus, modulates human NK cell activity. Reovirus activates NK cells in a type I interferon (IFN‐I) dependent manner, inducing STAT1 and STAT4 signalling in both CD56^dim^ and CD56^bright^ NK cell subsets. Gene expression profiling revealed the dominance of IFN‐I responses and identified induction of genes associated with NK cell cytotoxicity and cell cycle progression, with distinct responses in the CD56^dim^ and CD56^bright^ subsets. However, reovirus treatment inhibited IL‐15 induced NK cell proliferation in an IFN‐I dependent manner and was associated with reduced AKT signalling. *In vivo*, human CD56^dim^ and CD56^bright^ NK cells responded with similar kinetics to reovirus treatment, but CD56^bright^ NK cells were transiently lost from the peripheral circulation at the peak of the IFN‐I response, suggestive of their redistribution to secondary lymphoid tissue. Coupled with the direct, OV‐mediated killing of tumour cells, the activation of both CD56^dim^ and CD56^bright^ NK cells by antiviral pathways induces a spectrum of activity that includes the NK cell‐mediated killing of tumour cells and modulation of adaptive responses via the trafficking of IFN‐γ expressing CD56^bright^ NK cells to lymph nodes.

## Graphical abstract

Oncolytic reovirus treatment activates NK cells via a type I interferon‐dependent mechanism that modulates human NK cell activity in vitro and in vivo.

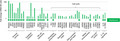

## INTRODUCTION

Natural killer (NK) cells are innate lymphoid cells (ILCs) with two broad functions, they detect and destroy infected cells and tumour cells and they provide signals for the initiation of adaptive immunity [1, 2]. Humans with NK cell defects are highly susceptible to viral infection [3, 4] and many viruses, herpesviruses and poxviruses in particular, encode numerous gene products that mediate evasion of NK cells [5, 6]. Indeed, battles between viruses and NK cells act as a major driver of both viral and NK cell evolution [7, 8]. Mouse and human studies have confirmed the importance of NK cells in anti‐tumour immunity [9] and the ability of NK cells to respond to both viral infection and cancer makes them a key mediator of the anti‐tumour activity of oncolytic viruses.

Oncolytic viruses (OV) are an emerging group of therapeutic agents [10, 11, 12, 13]. To date, one OV, Talimogene laherparepvec (T‐Vec; a derivative of Herpes Simplex Virus type I encoding GM‐CSF) has been approved for use in Europe and the USA for the treatment of advanced melanoma, and many more types of OV are in clinical trials in a range of cancers [14]. The therapeutic basis of OV activity was initially believed to result from their preferential replication in tumour cells, resulting in direct lysis [15, 16, 17]. However, it is now widely accepted that lytic activity is accompanied by the stimulation of anti‐tumour immunity, with NK cells playing a key role in their action. The use of OV enhances NK cell recruitment to tumours and depletion of NK cells (or NK cell activity) reduces the therapeutic effect of several OV types (rhabdoviruses, vaccinia virus and reovirus) in tumour‐bearing mice [18, 19, 20, 21, 22, 23]. Furthermore, clinical studies demonstrate that intravenous delivery of oncolytic Coxsackie A21 virus (in myeloid leukaemia) or Reovirus (in metastatic colorectal cancer) results in a type I interferon (IFN‐I) response and NK cell activation [24, 25]. Not surprisingly, the kinetics of NK cell activation by OV closely resemble those observed upon natural viral infection or vaccination [2, 25, 26].

Whilst many studies demonstrate that NK cells are critical to OV action, the anti‐viral activity of NK cells can be detrimental to their anti‐tumour effects, as reported in rodent models of liver and brain cancer [27, 28]. It is, therefore, of prime importance to investigate the mechanisms by which OV regulate the activity of NK cells. We have focussed our studies on the respiratory enteric orphan virus (Reovirus), a virus that normally causes asymptomatic infection in healthy individuals and one of the first viruses reported as having selectivity for tumour cells and proposed as a therapeutic agent [17]. Although reovirus encodes IFN‐I antagonists, the dsRNA genome is a powerful inducer of IFN‐I responses [13, 29]. The precise mechanisms underlying reovirus’ oncolytic activity are unclear, but likely result from the impairment of viral detection/IFN responses in malignant cells [13].

Studies to date have analysed OV action on bulk NK cell activity, with a focus on NK cell cytotoxicity [30, 31, 32]. However, human NK cells exist in two major subsets; CD56^dim^ NK cells predominate in the blood, they express CD16 and have strong cytotoxic activity, whereas the CD56^bright^ NK cell subset is less cytotoxic, have low (or no) CD16 expression and predominate in the secondary lymphoid tissue (SLT) [33, 34, 35]. Here, we have combined *in vitro* studies with the analysis of a reovirus clinical trial to gain insight into the action of OV on human NK cells. Our results demonstrate that oncolytic reovirus differentially affects the CD56^dim^ and CD56^bright^ NK cell subsets, modulating direct anti‐tumour effects, as well as responses that likely regulate adaptive immunity.

## MATERIALS AND METHODS

### Cells

Peripheral blood mononuclear cells (PBMC) were obtained from waste apheresis cones from healthy donors via NHS Blood and Transplant (UK). On the day of donation, PBMCs were separated by density gradient centrifugation using Lymphoprep (Axis‐Shield) and cultured in Roswell Park Memorial Institute medium (RPMI) 1640 (Sigma) +10% foetal calf serum (FCS) at a concentration of 2 × 10^6^ cells/ml at 37°C, 5% CO_2_. Natural killer (NK) cells were separated from PBMC, either on the day of donation or after the time specified, by negative immunomagnetic selection (Miltenyi Biotec). NK cells were cultured in Dulbecco's modified eagle medium (DMEM) supplemented with 10% human AB serum (Gemini Bio‐Products) and 10% FCS at 37°C, 5% CO_2_ unless otherwise specified.

### Reovirus and the clinical trial

Clinical grade reovirus (Pelareorep; formerly known as Reolysin; [13]) was provided by Oncolytics Inc. (Canada) and viral titres were determined by routine plaque assays on L929 cells. Overnight cultures of PBMC were treated with reovirus at a multiplicity of infection (MOI) of 1 (unless otherwise stated). For the trial, 10 patients with colorectal liver metastases were treated with intravenous reovirus prior to surgical resection of their tumour. Blood samples from reovirus‐treated patients were analysed immediately after collection. The clinical study (EUDRACT number 2007/000258‐29; [36]) was undertaken at the Leeds Cancer Centre following full ethical and regulatory approval. Patients were enrolled on the trial (and provided blood samples) following informed consent. The patient group and the clinical trial, including dose and scheduling of the reovirus treatment has been described previously [25, 36].

### Cytokine treatment

PBMC or NK cells were cultured overnight before treating with purified IFN‐α (Sigma) or recombinant IFN‐α (Miltenyi Biotec), recombinant IL‐12 (R&D systems and Peprotech) or recombinant IL‐15 (Miltenyi Biotec) as described in the text and figure legends. For IFN‐I neutralization, a cocktail of anti‐human interferon α/β receptor chain 2 antibody (clone MMHAR‐2), anti‐human interferon‐α (sheep polyclonal) and anti‐human interferon‐β (sheep polyclonal; all from PBL Assay Science) or a control cocktail of mouse IgG2a (BioLegend) and sheep serum (Sigma) was used, as described previously [25].

### Immunoblotting and ELISA assays

For immunoblotting, cells were washed with PBS and lysed in RIPA buffer with added protease and phosphatase inhibitors (Roche). Samples were sonicated, diluted in Laemmli sample buffer and separated by SDS‐PAGE before transferred to the PVDF membrane. Membranes were probed with antibodies listed in Table S1 and a secondary antibody conjugated to horseradish peroxidase, before developing using enhanced chemiluminescence substrate. For IFN‐I ELISA, 96 well plates were coated with a mixture of antibodies against IFN‐α (Mabtech, MT1/3/5) overnight at 4°C, and blocked with PBS supplemented with 10% FCS. Samples along with recombinant IFN‐α2 (Miltenyi), for construction of the standard curve, were added in triplicate and incubated overnight at 4°C. The plate was washed and IFN‐I assayed using a mixture of biotinylated detection antibodies against IFN‐α (Mabtech, MT2/4/6). Avidin‐conjugated alkaline phosphatase (Sigma, ExtrAvidin), and p‐Nitrophenyl phosphate substrate (Sigma, SigmaFast tablets) were used to develop the ELISA. Absorbance was read at 405 nm on a Multiskan EX plate reader (Thermo Fisher).

### Flow cytometry

For all flow cytometry experiments, staining buffer (PBS+2% FCS+0·09% sodium azide) was used for washing and staining steps. Isotype matched control antibodies were used in all experiments and a gate set whereby 2% of isotype control antibody‐stained cells were positive; for test antibodies, cells staining within this gate were assessed as positive. Cells were analysed on a LSRII flow cytometer (BD Biosciences) or a Cytoflex cytometer (Beckman Coulter). For cell sorting, an Influx cell sorter (BD Biosciences) was used. All antibodies used in flow cytometry experiments are listed in Table S1. NK cells within PBMC were identified as the CD56^+^CD3^neg^ population and additionally classified were required as CD56^bright^CD3^neg^ and CD56^dim^CD3^neg^ NK cells or as CD56^bright^CD16^low/neg^ or CD56^dim^CD16^+^ cells; gates for identification of NK cells for analytical or sorting approaches are shown in Figure S1.

### Intracellular staining

For granzyme B, cells were first stained for surface proteins, fixed in Cytofix buffer (BD Biosciences), washed and incubated in saponin buffer (staining buffer +0·1% saponin), and stained in saponin buffer plus anti‐granzyme B antibody (or a matched isotype control). Cells were resuspended in 0·5% paraformaldehyde (in staining buffer) prior to analysis. For other intracellular proteins, cells were stained for surface proteins if required and then fixed in Cytofix fixation buffer (BD Biosciences), according to the manufacturer's instructions. For time‐course experiments, fixed cells were stored at 4°C and all cells within an experiment were stained at the same time. Samples were then resuspended in permeabilization buffer III (BD Biosciences) and permeabilized on ice for 30 min, followed by staining, washing and analysis.

### NK cell degranulation assay

PBMC were co‐cultured with K562 cells in a 96 well, round‐bottomed cell culture plate (Corning), at an effector:target ratio of 10:1. After 1 h of culture, GolgiStop (BD Biosciences) was added and cells were cultured for a further 5 h before staining for NK cell markers and cell surface CD107a.

### Proliferation and cell cycle profiling

For CFSE labelling, cells were labelled by suspension in warm PBS, containing 2 μM CFDA‐SE (Invitrogen) and incubation for 10 min at 37°C. The labelling reaction was quenched with an equal volume of warm FCS and analysed by flow cytometry. For propidium iodide (PI) staining, washed cells were resuspended in ice‐cold 70% ethanol at 1 × 10^6^ cells/ml with vortexing. Samples were fixed on ice for 30 min then stored at −20°C. For staining, fixed cells were washed with 2 ml stain buffer, centrifuging at 600x*g*. Samples (0·5–1 × 10^6^ cells) were resuspended in 50 μl of 100 μg/ml RNase A (Qiagen). PI (Life Technologies) at 16·6 μg/ml in staining buffer was added directly to samples in RNase A (final density of 10^6^ cells in 650 μl). Samples were incubated at room temperature for 10 min and analysed using an LSRII flow cytometer (BD Biosciences), on the lowest speed setting. Cell cycle profiling was performed using Modfit (Verity software) according to the manufacturer's recommendations.

### Gene expression profiling and data analysis

PBMC isolated from five healthy donors were cultured with or without 1 MOI reovirus for 48 h. NK cells were isolated by immunomagnetic selection and resuspended in RNAprotect Cell Reagent (Qiagen); RNA was extracted with the RNeasy mini kit (Qiagen), according to the manufacturer's instructions. Contaminating DNA was removed by on‐column DNase digestion and RNA integrity was checked using an Agilent 2100 Bioanalyzer (Agilent Technologies). The total RNA was amplified, sense strand cDNA synthesized and labelled using the GeneChip™ WT PLUS Reagent Kit (Applied Biosystems, Thermo Fisher Scientific). Labelled cDNA was hybridized to an Affymetrix GeneChip^®^ Human Transcriptome Array 2·0 (Applied Biosystems, Thermo Fisher Scientific). Raw intensity files (CEL files) for all conditions were processed with the Expression Console software (Affymetrix) using the Signal Space Transformation‐Robust Multi‐Chip Analysis (SST‐RMA) algorithm. Normalized signal values were analysed with the Transcriptome Analysis Console (TAC, Affymetrix) software, to identify statistically significant differences between conditions. Following the manufacturer's guidelines, TAC software was used to run paired ANOVA tests and false discovery rate (FDR) prediction. Differentially expressed genes were defined as >1·5 fold up or downregulated with FDR <0·05. Gene set enrichment analysis was performed using the tools provided by Enrichr [37, 38]; available at https://amp.pharm.mssm.edu/Enrichr/. Interferon regulation of genes was analysed using the interferome database [39]; available at http://www.interferome.org/interferome/home.jspx. We used v2·01 of the database and restricted our analysis to human genes regulated (>1·5 fold change) by IFN‐I in haematopoietic cells, using the filters provided. To analyse the intersection of datasets we used the Venn diagram drawing tool available at; http://bioinformatics.psb.ugent.be/webtools/Venn/. The microarray data are available at the EMBL‐EBI Array Express repository (https://www.ebi.ac.uk/arrayexpress/) with the accession number E‐MTAB‐9826.

### Quantitative RT‐PCR

Cells were resuspended and stored in RNAprotect cell reagent (Qiagen). RNA was extracted with the RNeasy mini kit (Qiagen) and contaminating DNA removed by on‐column DNase digestion, with the RNase‐Free DNase Set (Qiagen). cDNA was synthesized using random primers (New England Biolabs) and the Superscript III reverse transcriptase kit (Invitrogen). qPCR amplification was performed with either Taqman or SYBR Green reagents. For both, 10ng cDNA was used together with assay‐specific primers (Table S2), and either PowerUp™ SYBR™ Green Master Mix (for SYBR Green method) or Taqman gene expression Mastermix (both Applied Biosystems). Amplification was carried out in a 7500 real‐time PCR machine or a QuantStudio 5 machine (Applied Biosystems). Reactions were performed in triplicate, apart from the qPCR of cell sorted CD56^dim^ and CD56^bright^ subsets where there was limited starting material and reactions were performed in duplicate. All technical replicate values were within 0·5 cycles. Fold change gene expression was calculated by the ΔΔCt method [40], normalizing to ABL1 as the housekeeping gene.

### Statistical testing

Tests (identified in the figure legends) were performed using Prism (GraphPad Software).

## RESULTS

**FIGURE 1:**
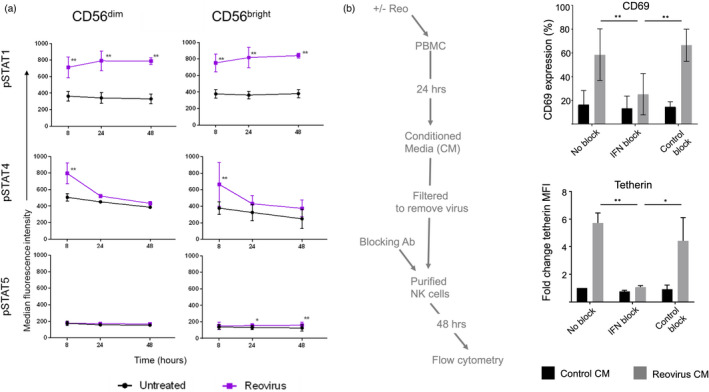
STAT phosphorylation and IFN‐I dependent NK cell activation following reovirus treatment. (a) STAT phosphorylation in CD56^bright^ and CD56^dim^ NK cells (detected by intracellular staining and flow cytometry) in PBMC cultured without virus (untreated; black line) or with 1 MOI reovirus (purple line), for 8, 24 and 48 h. Graphs show mean MFI and standard deviation from three donors. Data were analysed by two‐way repeated‐measures ANOVA, followed by Sidak multiple comparisons test. *p < 0·05 **p < 0·01. (b) NK cell activation by reovirus is IFN‐I dependent. The flow chart shows the approach taken; PBMC (from one donor) were left untreated or treated with reovirus for 24 h and the conditioned media (CM) filtered to remove viruses. CM was added to purified NK cells in the presence of an IFN‐I blocking antibody cocktail (IFN block), a control blocking cocktail (control block) or no added antibody (no block). CM from untreated PBMC was used a control. After 48 h, the NK cell surface expression of CD69 and tetherin was measured by flow cytometry. Data is from control CM or CM from reovirus‐treated PBMC from a single donor, applied to three NK cell donors. The *y*‐axes show the percentage of CD69 expressing cells (top panel) or the fold change in MFI of tetherin relative to control CM and no added antibody treatment (bottom panel), due to constitutive low‐level expression of this molecule on unstimulated NK cells [25]. Differences between mean percentage positive values for CD69, or mean fold change MFI for tetherin, were analysed by two‐way repeated‐measures ANOVA, followed by Sidak multiple comparisons test. *p < 0·05 **p < 0·01

Our clinical trials of reovirus in colorectal cancer and glioblastoma have utilized intravenous (IV) delivery [36, 41]. Blood NK cell activation in reovirus‐treated patients occurs 24–48 h post‐virus infusion and is co‐incident with the peak of a type I interferon (IFN‐I) response [25]. However, several cytokines are implicated in the virus‐dependent activation of NK cell activity. In particular, IFN‐I, IL‐12 and IL‐15 have established roles in viral infection and NK activation [42, 43]. Signal transduction pathways for these cytokines overlap, but they utilize characteristic JAK/STAT pathways that can be used to indicate their activity; IFN‐I signals predominantly via STAT1 and STAT4, IL‐12 via STAT4 and IL‐15 via STAT5 [44, 45, 46, 47]. To model IV‐delivery *ex vivo*, we treated PBMC from healthy donors with reovirus at a multiplicity of infection (MOI) of 1 (approximating the dose used in the clinical trials) and analysed STAT phosphorylation by intracellular staining and flow cytometry in CD56^bright^ and CD56^dim^ NK cells at 8, 24 and 48 h post‐treatment, reasoning that cytokines induced during treatment would take time to accumulate. We determined the patterns of STAT phosphorylation in the NK cells from reovirus‐treated PBMC (Figure 1a) and compared these to STAT phosphorylation found in purified NK cells and PBMC treated with cytokines (Figure S2a–d). Statistically significant phosphorylation of STAT5 was detected in CD56^bright^ NK cells in response to reovirus (Figure 1a), but this was a very small effect compared to that observed with both the CD56^bright^ and CD56^dim^ NK cell subsets treated with IL‐15 (Figure S2a–d) and it is unlikely that such a small change in STAT5 phosphorylation has biological significance. In contrast, both the CD56^dim^ and CD56^bright^ NK cell subsets showed clear activation of both the STAT1 and STAT4 pathways following reovirus treatment of PBMC (Figure 1a). These data are consistent with IFN‐I induced signalling in both CD56^bright^ and CD56^dim^ NK cells and, as expected, IFN‐I was readily detectable in the supernatants of reovirus treated PBMC (Figure S2e). We confirmed the importance of this IFN‐I in reovirus‐mediated NK cell activation. Reovirus‐conditioned media (rCM) from PBMC treated with reovirus for 24 h was collected, filtered to remove virus particles and added to purified NK cells for 48 h in the presence or absence of anti‐IFN antibodies (the approach is summarized in Figure 1b); rCM‐induced cell surface expression of CD69, a marker of NK cell activation, and tetherin, an IFN‐induced antiviral protein, on purified NK cells in an IFN‐I dependent manner (Figure 1b). These data suggest that the IFN‐I‐dependent NK cell activation observed in response to reovirus is associated with the direct action of IFN‐I on the NK cells themselves, with STAT1 and STAT4 phosphorylation readily detectable in both CD56^dim^ and CD56^bright^ NK cell subsets.

**FIGURE 2:**
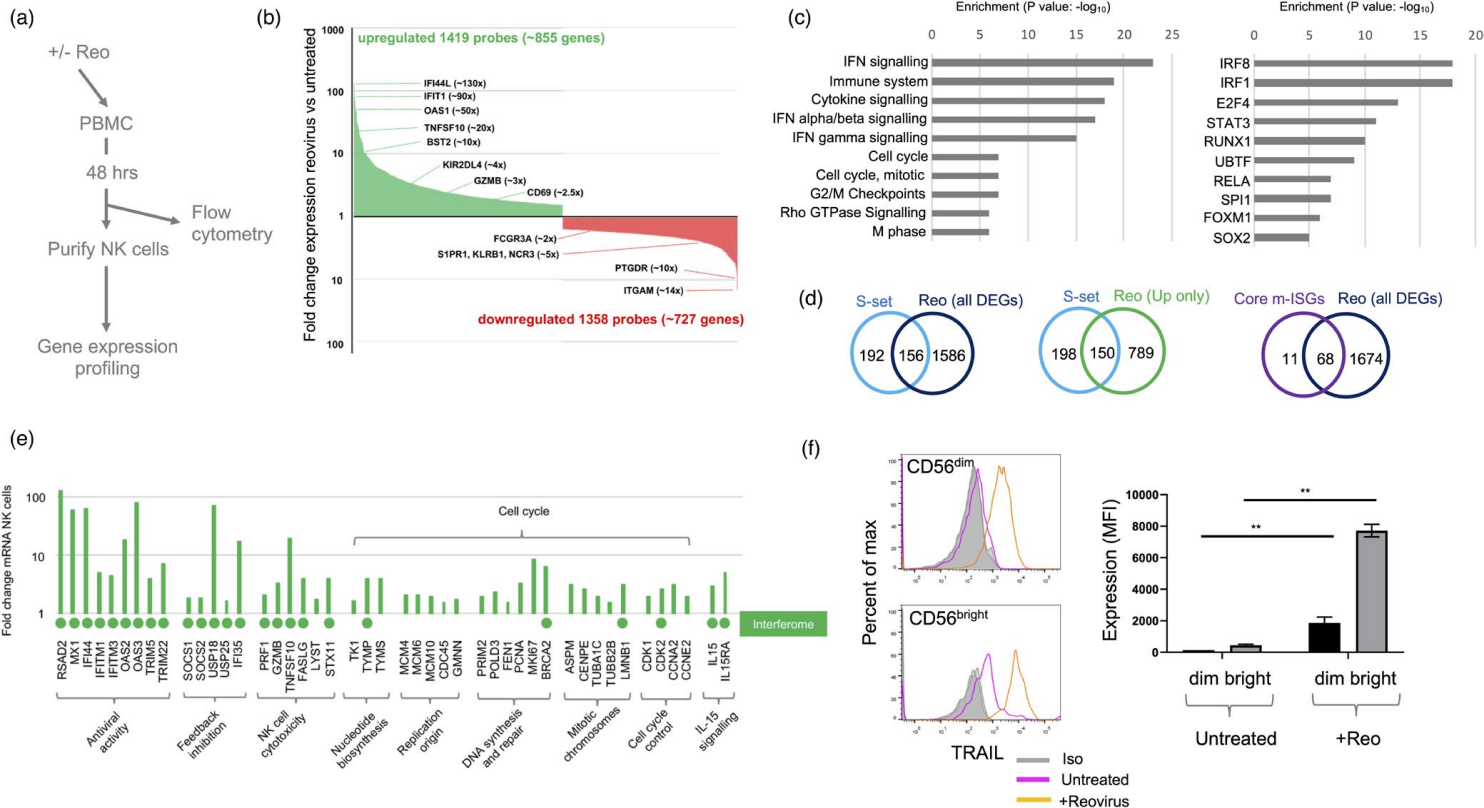
Gene expression profiling of NK cells following reovirus treatment. (a) Summary of the approach. PBMC from five healthy donors were treated at an MOI of 1 with reovirus (or left untreated). After 48 h, NK cells were purified using immunomagnetic selection and a small aliquot of cells was removed for flow cytometry to assess CD69 induction (Figure S3). The remainder of the NK cells were used in the gene expression profiling. (b) Differentially expressed genes in total human NK cells following reovirus treatment. Genes that were up (green columns) or downregulated (red columns) together with examples and fold change are indicated. Data underlying this graph is shown in Table S3. (c) Gene set enrichment analysis (GSEA). The left panel shows the top 10 pathways identified by GSEA of differentially expressed genes. Data were generated using Enrichr and the output from Reactome 2016; the adjusted *p*‐value of enrichment is shown. Full data is provided in Table S4. The right panel shows the top 10 transcription factors associated with differentially expressed genes. Data were generated using Enrichr and the output from ENCODE and ChEA Consensus Transcription factors from ChIP‐X; the adjusted *p*‐value of enrichment is shown. Full data are provided in Table S5. (d) Venn diagrams showing the overlap of the differentially expressed genes (DEGs) from NK cells following reovirus treatment (Reo) with the interferon‐stimulated genes listed in Schoggins et al ([50]; S‐Set) or the core mammalian ISGs identified by Shaw et al ([51]; Core m‐ISGs). Overlaps were determined for all reovirus DEGs (left and right panels) or the upregulated genes only (centre panel). (e) Expression of genes from selected pathways. The graph shows the fold change in gene expression in NK cells following reovirus treatment, with genes and pathways indicated. A green circle below the *x*‐axis indicates that the gene is induced by IFN‐I in haematopoietic cells, as determined using data from the Interferome database [39]. (f) TRAIL expression by CD56^dim^ and CD56^bright^ NK cells following reovirus treatment. The histograms show flow cytometry from a single representative donor, with the different treatments (and isotype control antibody) indicated, along with the NK cell subset analysed via gating. The graphs on the right show the mean and standard deviation of the median fluorescence intensity (MFI), from three separate donors; data were analysed by a repeated‐measures one‐way ANOVA, with Tukey's multiple comparison test; **p < 0·01

To further explore the responses of NK cells to reovirus treatment, we performed gene expression profiling, as summarized in Figure 2a. We treated PBMC from five healthy donors with reovirus for 48 h and analysed a small sample by flow cytometry; NK cells from all donors upregulated cell surface expression of CD69 in response to reovirus (Figure S3). We purified the NK cells from the remaining PBMC and performed gene expression profiling by microarray. Differentially regulated transcripts were detected by 2777 probes, representing some 1742 genes, with an approximately equal number of genes induced and repressed by reovirus treatment (Figure 2b and Table S3). Amongst the upregulated genes were IFN stimulated genes (ISGs), including IFI44L and IFIT1, which were also induced in NK cells following reovirus treatment of cancer patients [25]. Similarly, BST2 (encoding tetherin) and CD69 were induced at the mRNA level, consistent with detection of their protein products at the cell surface following reovirus treatment in vitro (Figure 1b) and in vivo [25]. Genes whose expression was downregulated by reovirus treatment included FCGR3A (encoding CD16), the NK cell receptors KLRB1 (NKR‐P1; CD161) and NCR3 (NKp30; CD337) and the sphingosine‐1‐phosphate receptor, S1PR (Figure 2b). We used gene set enrichment analysis (GSEA) to analyse the effects of reovirus on NK cells in more detail, exploiting the multiple tools available via the Enrichr suite [37, 38]. As expected, GSEA identified IFN regulated pathways as the most highly enriched amongst 1742 differentially expressed genes but also highlighted enrichment of genes regulating the cell cycle (Figure 2c and Table S4). Accordingly, transcription factors associated with the differentially expressed genes included mediators of IFN and inflammatory responses, as well as regulators of the cell cycle (Figure 2c) [48, 49].

We analysed the interferon response in more detail. We compared our data with a published list of 348 ISGs [50]; 156 of these 348 ISGs were differentially regulated, with 150 of the 156 genes being induced in NK cells during reovirus treatment of PBMC (Figure 2d). In addition, we compared the genes upregulated in NK cells with a set of 79 ISGs induced across nine species of mammals, termed core mammalian ISGs [51]; four‐fifths of these core ISGs, encoding antiviral and other IFN‐I regulated functions were upregulated in the human NK cells following reovirus treatment of PBMC (Figure 2d). The action of IFN‐I includes the induction of feedback inhibitory pathways which limit responses [52]. In our previous clinical trial, most patients received multiple infusions of reovirus yet only demonstrated NK cell activation after the first virus infusion [25]. We speculated that this was due, at least in part, to the induction of feedback inhibitory mechanisms that limit IFN‐I responses. We analysed the gene expression profiling for induction of candidate feedback inhibitors and found that the IFN‐I signalling antagonists, SOCS1, SOCS2, USP18, USP25, and IFI35 were all induced in NK cells following reovirus treatment of PBMC (Figure 2e).

We previously showed that reovirus activates NK cell cytotoxicity in an IFN‐I dependent manner [31]. The expression profiling performed here identified several genes encoding cytotoxic effector molecules, namely PRF1 (perforin), GZMB (granzyme B), TNFSF10 (TRAIL), and FASLG (Fas ligand) as being upregulated in NK cells by reovirus treatment, along with two genes, LYST (lysosomal trafficking regulator) and STX11 (syntaxin 11), which are critical in the biosynthesis and exocytosis of cytotoxic granules [53] (Figure 2e). The 20‐fold induction of TNFSF10 mRNA was mirrored by induction of TRAIL on the NK cell surface; in the absence of stimulation, TRAIL was expressed at low levels on CD56^bright^ NK cells and was undetectable on the CD56^dim^ NK cells. However, reovirus treatment significantly induced cell surface TRAIL expression on both subsets (Figure 2f). Furthermore, the induction of cell surface TRAIL by reovirus exceeded that found with IL‐15 (Figure S4a). For the granule‐mediated cytotoxic pathway, NK cells showed enhanced degranulation in response to tumour target cells (Figure S4b), confirming our previous data [25]. We analysed intracellular granzyme B levels using flow cytometry and found that the cytotoxic CD56^dim^ NK cell subsets showed little change in granzyme B content, whereas the CD56^bright^ NK cells, which have low cytotoxic activity under resting conditions, did induce expression of granzyme B upon reovirus treatment (Figure S4c). Treatment with IFN‐I itself induced a modest increase in granzyme B in purified NK cells and significantly enhanced granule exocytosis, albeit to a lesser extent than IL‐15 (Figure S4d,e). These data reveal that reovirus induces NK cell cytotoxic molecules in both CD56^dim^ and CD56^bright^ NK cell subsets.

Our GSEA also revealed the induction of genes associated with cell cycle progression (Figure 2c,e; Table S4), suggesting that reovirus treatment might induce NK cell proliferation. Indeed, studies performed in mice suggest that IFN‐I responses induce the expression of IL‐15 (e.g. in dendritic cells) and that this, in turn, activates NK cell cytotoxicity and proliferation [42, 43]. Gene expression profiling identified induction of the IL‐15 receptor ⍺ chain gene (IL15RA) in NK cells, as well as increased expression of IL15 itself (Figure 2e). The transcriptional programme downstream of reovirus treatment is clearly dominated by IFN‐I responses. However, a variety of stimuli were expected to contribute to the patterns of gene expression observed in NK cells from reovirus treated PBMC. We used the interferome database [39] to assess induction of the 45 genes shown in Figure 2e in response to IFN‐I treatment; IFN‐I induces genes encoding antiviral function, feedback inhibition of IFN‐I responses, NK cell cytotoxicity and IL‐15/IL‐15R⍺, but the majority of the cell cycle functions analysed were not induced by IFN‐I in haematopoietic cells (Figure 2e).

**FIGURE 3:**
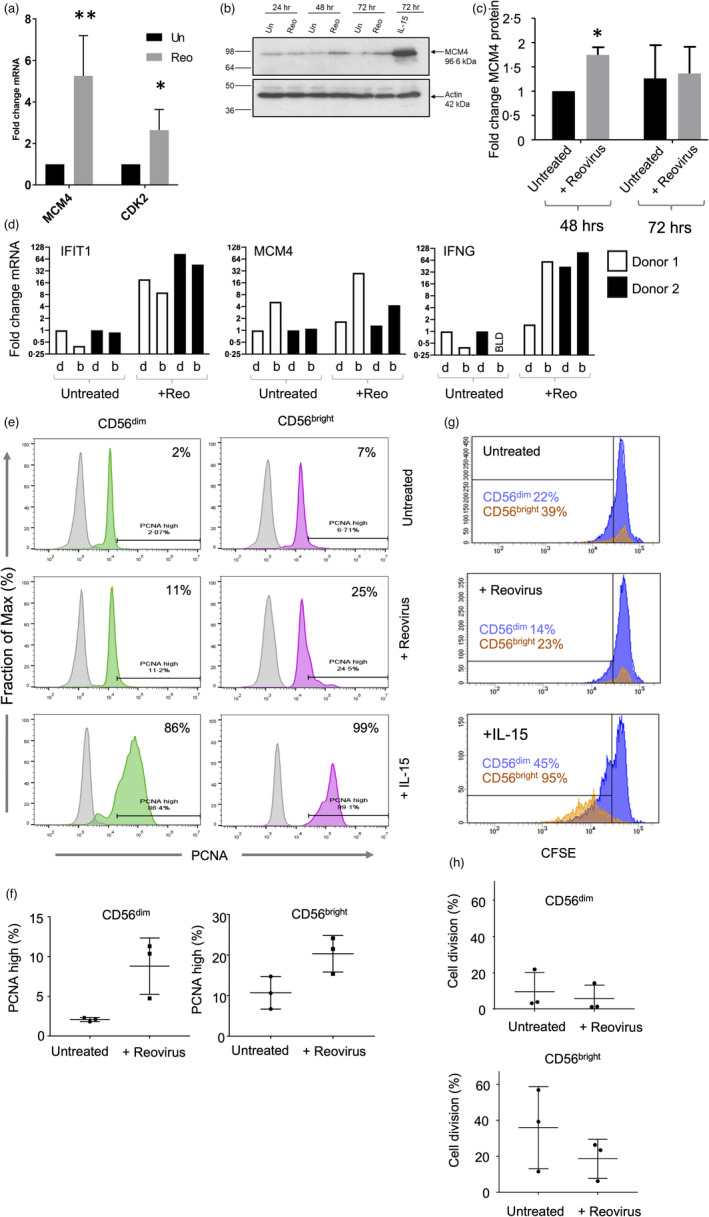
Cell cycle analysis in NK cells following reovirus treatment. (a) Induction of MCM4 and CDK2 genes in NK cells following reovirus treatment. PBMC were treated with reovirus for 48 h, NK cells purified and gene expression determined by qRT‐PCR (in three or four donors respectively); mean fold change relative to untreated in each donor with standard deviation shown. Data were analysed by two‐way repeated‐measures ANOVA, followed by Sidak multiple comparisons test. *p < 0·05 **p < 0·01. (b) Time course of MCM4 protein expression in NK cells following reovirus treatment. PBMC were cultured with or without 1 MOI reovirus, or with 10 ng/ml IL‐15, for either 24, 48 or 72 h after which NK cells were purified. Protein expression of MCM4 (and β‐actin) was analysed in NK cells by immunoblotting; the data shown are from a single donor. (c) Quantitation of immunoblotting from three donors, with fold change in MCM4 expression determined by comparison to β actin. Mean fold change and standard deviation are shown, analysed by *t*‐test; *p < 0·05. (d) Expression of MCM4, IFNG and IFIT1 genes in CD56^dim^ (d) and CD56^bright^ (b) NK cells in NK cells from untreated or reovirus treated PBMC. Following 48 h of reovirus treatment of PBMC, CD56^dim^ and CD56^bright^ subsets were purified by cell sorting and gene expression determined by qRT‐PCR. Expression is shown relative to the untreated controls, as indicated. Data are shown separately for two donors (indicated by shading). Cell sorting gates are shown in Figure S1C. (e) PCNA expression in CD56^dim^ and CD56^bright^ NK cells following reovirus treatment. PBMC were cultured with or without reovirus, or with 10 ng/ml IL‐15, for 48 h and PCNA expression was analysed by intracellular staining and flow cytometry. Data from a single donor is shown with the PCNA high gate indicated (set as the top 2% of untreated CD56^dim^ cells) along with the percentage of PCNA high expressing cells. (f) Mean percentage of PCNA high cells in CD56^dim^ and CD56^bright^ NK cells (determined as in e) from three donors. Analysis by paired t‐test was not statistically significant. (g) Cell division following reovirus treatment. PBMC were labelled with CFSE and cultured with or without 1 MOI reovirus, or 10 ng/ml IL‐15. After 5 days, CFSE content in CD56^dim^ and CD56^bright^ NK cells was determined by flow cytometry. The percentage of cells with reduced CFSE content due to cell division are indicated. These data are from a single donor. (h) Proliferation of CD56^dim^ and CD56^bright^ NK cells from the CFSE assay was performed in three separate donors (as in g). Differences between treatment means were tested using a paired t‐test and were not statistically significant

**FIGURE 4:**
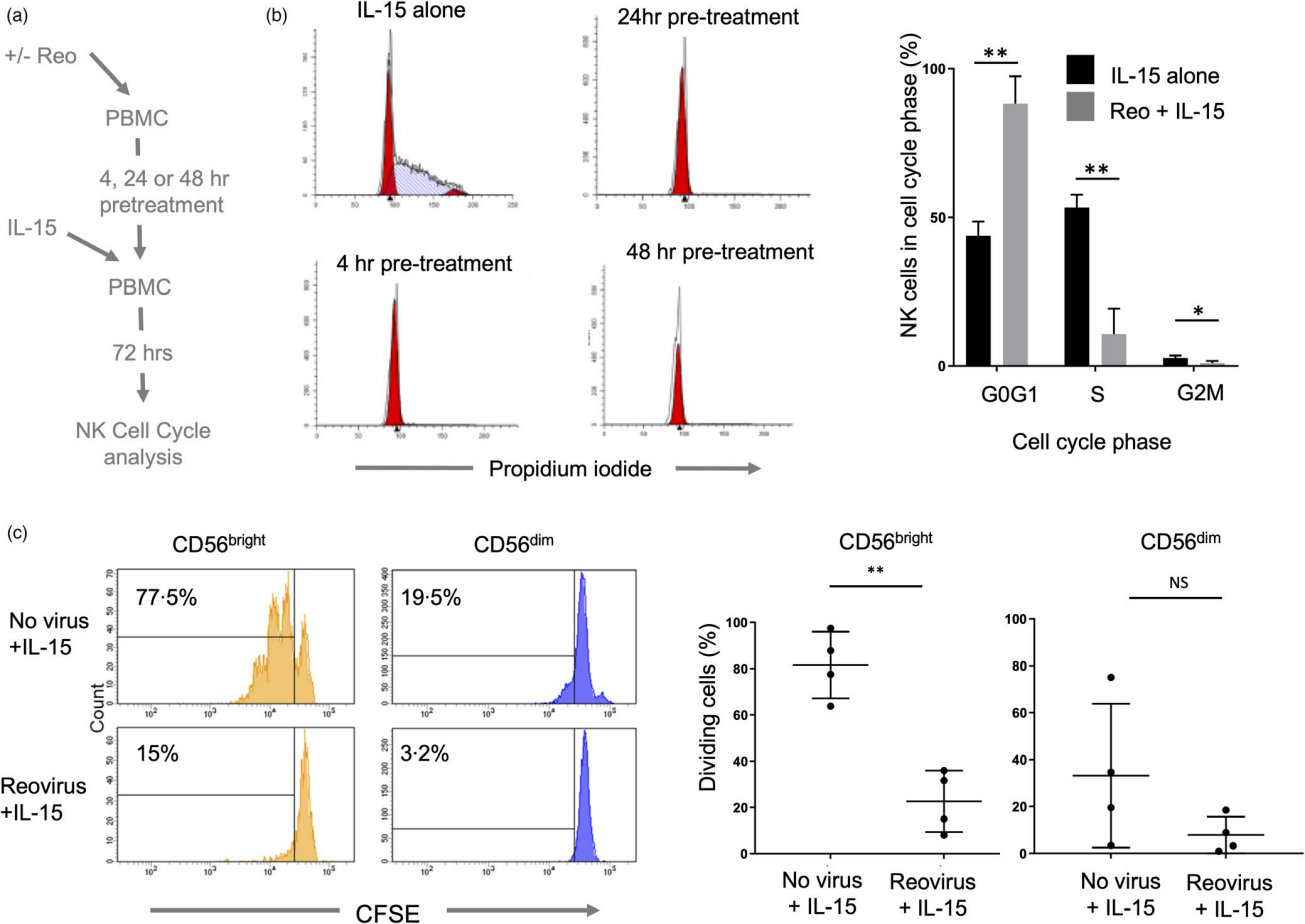
Inhibition of NK cell proliferation following reovirus treatment. (a) Summary of the approach. PBMC were pre‐treated with/without reovirus for 4, 24 or 48 h and IL‐15 (10 ng/ml) was then added for 72 h, after which cell cycle profiling was performed. (b) Cell cycle profiling by DNA content. PBMC were treated with either 10 ng/ml IL‐15 for 72 h, or pre‐treated with 1 MOI reovirus for 4, 24 or 48 h, followed by IL‐15 treatment for 72 h. NK cells were purified, fixed and DNA stained with propidium iodide and analysed by flow cytometry. Cell cycle profiles were analysed using Modfit software to quantify the percentage of cells in each compartment, as shown in the graph (collated from three donors). Shown are the mean and standard deviation of percentages. Differences between percentages in each compartment were tested by multiple unpaired *t*‐tests, with corrections for multiple tests by the Holm‐Sidak method. **adjusted *p* < 0·01, *adjusted *p* < 0·05. (c) Reovirus mediated inhibition of IL‐15 induced NK cell proliferation. PBMC were labelled with CFSE, pre‐treated for 4 h with 1 MOI reovirus or cultured without virus. 10 ng/ml IL‐15 was then added and cells were cultured for a further 5 days. Cell division was assessed by CFSE loss using flow cytometry. Plots from a single donor are shown on the left with the percentage of cells that have proliferated after 5 days indicated. Data from four donors are shown on the right, analysed using a *t*‐test; **p < 0·01. NS = not significant

We investigated the induction of cell cycle characteristics in more detail. Reovirus‐mediated induction of genes encoding positive regulators of the cell cycle (MCM4 and CDK2) was confirmed using qRT‐PCR (Figure 3a). Induction of MCM4 mRNA was associated with a significant increase in MCM4 protein 48 h post‐reovirus treatment (Figure 3b,c). Cell sorting and qRT‐PCR showed that reovirus induced the expression of the IFIT1, IFNG and MCM4 genes, with MCM4 preferentially induced in the CD56^bright^ NK cell subset in two donors analysed (Figure 3d). The induction of cell cycle‐related molecules was suggestive of NK cell proliferation. Markers of proliferating cells include proliferating cell nuclear antigen (PCNA) and Ki67. At the protein level, reovirus‐mediated induction of PCNA in NK cells was much weaker than that observed with IL‐15. Nevertheless, reovirus did induce PCNA expression in both CD56^dim^ and CD56^bright^ NK cells (albeit not reaching statistical significance), with greater induction in the latter subset (Figure 3e,f). Similar experiments analysing Ki67 expression failed to show induction of this marker in either NK cell subset following reovirus treatment, although expression in response to IL‐15 was observed (Figure S5). However, despite the proliferative signature, reovirus treatment of PBMC did not induce division of either the CD56^dim^ or CD56^bright^ NK cells, as judged using a 5‐day CFSE assay (Figure 3g,h). This was in sharp contrast to the potent mitogenic activity of IL‐15, with the CD56^bright^ NK cells being more responsive to IL‐15 than the CD56^dim^ subset (Figure 3g), consistent with previous data [54]. These results suggested that reovirus treatment of PBMC was inducing a number of components associated with proliferation in NK cells, but that the signals delivered were either insufficient to initiate proliferation and/or were being counteracted by anti‐proliferative effects,

The induction of the IL15RA gene encoding the high‐affinity α‐subunit of the IL‐15 receptor suggested that reovirus treatment might prime NK cells for subsequent proliferative responses, as shown using mouse models and other viruses [42, 43]. We pre‐treated PBMC with reovirus for 4, 24 or 48 h followed by IL‐15 for a further 3 days and analysed the cell cycle profile (Figure 4a). The addition of IL‐15 alone induced the S phase, but pre‐treatment with reovirus for as little as 4 h caused a block in cell cycle progression (Figure 4b). Furthermore, pre‐treatment with reovirus blocked the IL‐15‐mediated proliferation of both the CD56^dim^ and CD56^bright^ subsets, with statistically significant impairment of the more proliferative CD56^bright^ NK cells (Figure 4c).

**FIGURE 5:**
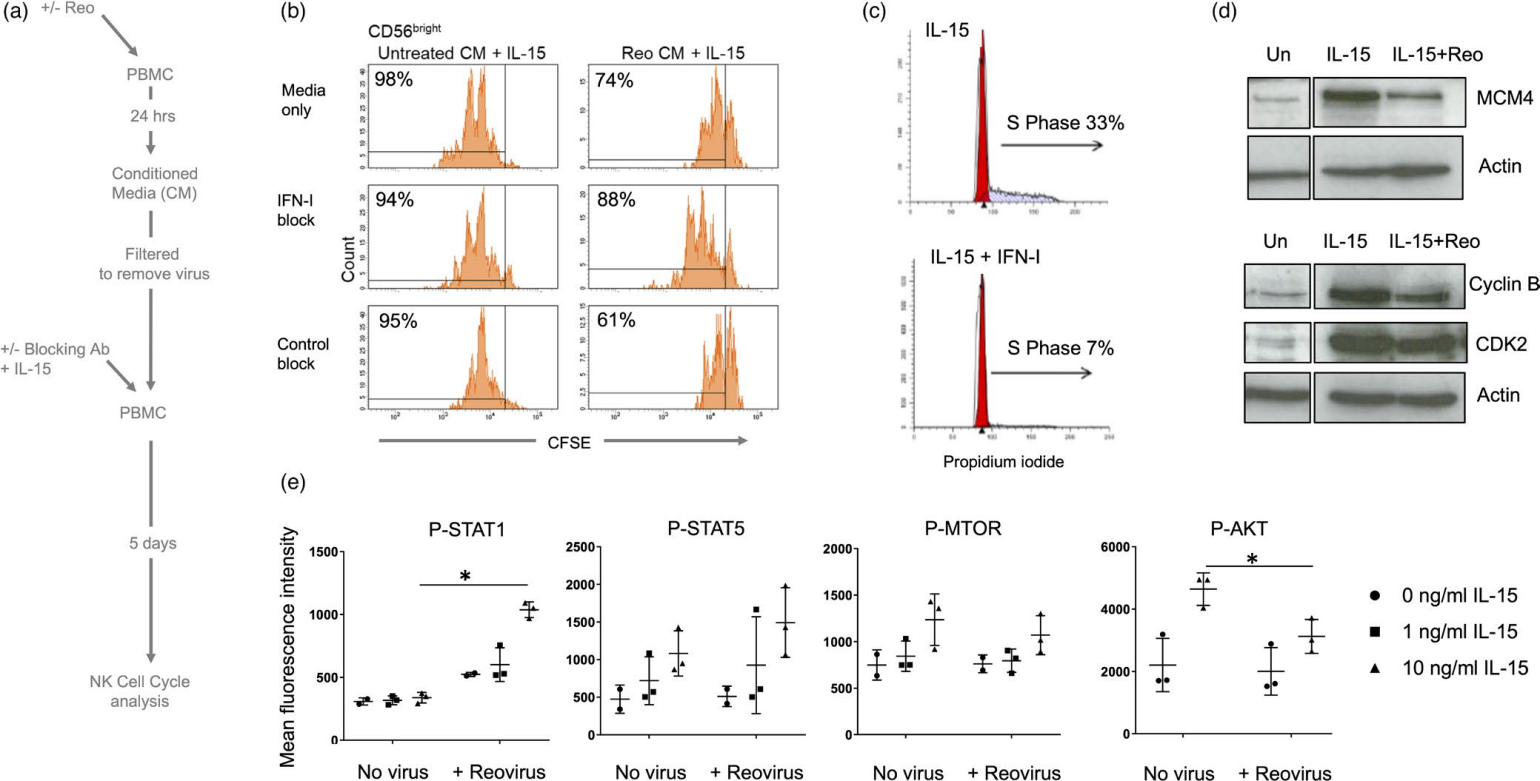
Reovirus‐induced IFN‐I production inhibits IL‐15‐mediated proliferation of NK cells. (a) Summary of the approach; Conditioned media (CM) was collected from reovirus treated (or untreated) PBMC and filtered to remove virions. This CM was applied to fresh PBMC (labelled with CFSE) together with IL‐15, in the presence or absence of IFN‐I blocking antibodies. PBMC were cultured for 5 days and proliferation of CD56^bright^ NK cells analysed by CFSE content. (b) Inhibition of CD56^bright^ NK cell proliferation by reovirus‐induced IFN‐I. CFSE analysis of CD56^brigh^t NK cells following treatment described in (a). The percentage of cells that have proliferated after 5 days are indicated. These data are from a single donor, representative of three donors. (c) IFN‐I blocks IL‐15 induced S phase. Purified NK cells were cultured with IL‐15 or IL‐15+IFN‐I (all at 100 ng/ml) for 3 days and S phase assessed by propidium iodide staining, as shown in panel (a). This data is from one donor, representative of three donors tested. (d) Reovirus treatment blocks IL‐15 induced expression of cell cycle mediators. PBMC were cultured for 4 h alone or primed with 1 MOI reovirus (Reo). 10 ng/ml IL‐15 was then added directly to all samples for 3 days. Total NK cells were isolated and MCM4, cyclin B and CDK2 analysed by immunoblotting along with β‐actin as a loading control. The blot image has been cut between the unstimulated control and cytokine treated lanes as shown by the boxing. These data are representative of three donors tested. (e) Modulation of IL‐15 mediated signalling by reovirus treatment. PBMC were cultured for 48 h alone (no virus) or primed with 1 MOI reovirus (+reovirus). After 48 h, 0, 1 or 10 ng/ml IL‐15 was added (as indicated) for 30 min. Phospho‐STAT1, STAT3, STAT5, mTOR and Akt were analysed by intracellular staining and flow cytometry, gating on the NK cell population. Graphs show median fluorescence intensities (MFI) for two or three separate donors, with standard deviation. Data were analysed by two‐way repeated‐measures ANOVA. When the effect of the virus was statistically significant, a post hoc Sidak multiple comparison tests was applied to identify statistically significant differences between “no virus” and “reovirus” MFI values; *p < 0·05

We analysed the role of reovirus‐induced IFN‐I in the inhibition of IL‐15‐mediated proliferation, focussing on the highly proliferative CD56^bright^ NK cell subset. The approach is summarized in Figure 5a. Conditioned media was collected from reovirus treated PBMC and filtered to remove virions. This CM was applied to fresh PBMC together with IL‐15, in the presence or absence of IFN‐I blocking antibodies. This revealed that, for CD56^bright^ NK cells, the inhibition of IL‐15 mediated proliferation observed with rCM was IFN‐I dependent (Figure 5b). This was confirmed by stimulating purified NK cells with IL‐15 or IL‐15 plus IFN‐I, showing that IFN‐I reduced the mitogenic activity of IL‐15, as determined by cell cycle profiling (Figure 5c). Furthermore, immunoblotting showed that pre‐treatment with reovirus reduced the IL‐15 mediated induction of MCM4, cyclin B and CDK2 (Figure 5d). The ability of reovirus treatment (and IFN‐I) to block IL‐15 mediated proliferation of NK cells suggested that IL‐15 mediated signalling events might be disrupted. We treated PBMC with reovirus for 48 h then added IL‐15 for 30 min and analysed the phosphorylation of STAT1, STAT5, mTOR, and AKT in NK cells using intracellular flow cytometry. The IL‐15 mediated phosphorylation of STAT5 and mTOR was unaffected by pre‐treatment with reovirus, but IL‐15‐induced AKT phosphorylation was significantly reduced (Figure 5e). Reovirus pre‐treatment also enhanced STAT1 phosphorylation downstream of IL‐15, a pathway not typically activated by this cytokine (Figure 5e). Taken together, these data show that reovirus primed NK cells have skewed signalling pathway activation and reduced proliferation in response to IL‐15, with the reovirus induced IFN‐I counteracting the mitogenic activity of IL‐15.

**FIGURE 6:**
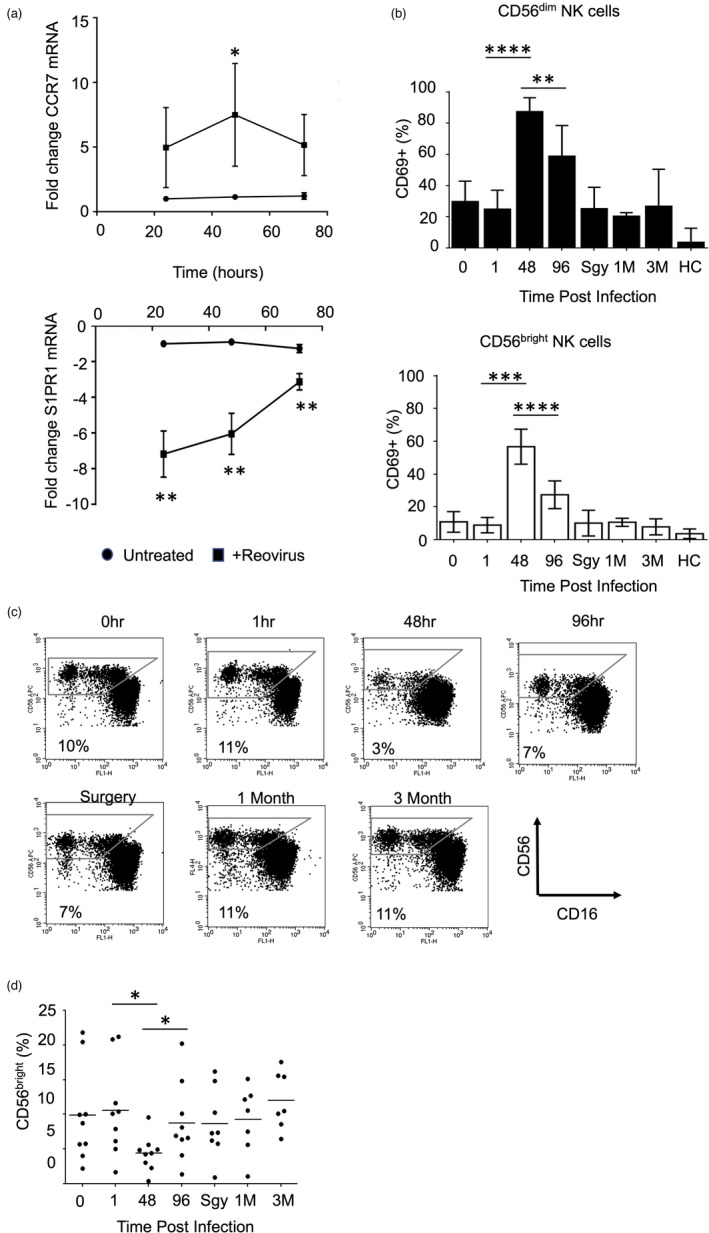
Reovirus treatment alters human NK cell subset distribution in vivo. (a) Expression of CCR7 and S1PR1 genes in NK cells following reovirus treatment in vitro. PBMC were treated with reovirus for 24, 48 and 72 h, NK cells were isolated and gene expression assessed using qRT‐PCR. The data show the fold change in expression in reovirus treated cells compared to untreated, indicating the mean and standard deviation from three donors. The effect of time and treatment on mean ddCt values was tested by two‐way repeated‐measures ANOVA, followed by Sidak multiple comparisons test; *p < 0·05, **p < 0·01. (b) NK cell CD69 expression following reovirus treatment of patients. Nine cancer patients were treated with intravenous reovirus at time zero with blood samples taken immediately prior to infusion (0 h) and at 1, 48 h and 96 post‐infusion. Patients had another blood sample taken immediately prior to surgery (Sgy) 1–4 weeks after infusion and additional samples 1 month (1 M) and 3 months (3 M) later. Samples were also taken from untreated healthy controls (HC); full details of the patients, controls and treatment scheduling have been published previously [25]. Blood samples were used to assess NK cell activation by cell surface CD69 expression on the CD56^dim^ (top panel) and CD56^bright^ (bottom panel) NK cell subsets. Statistically significant changes in CD69 expression, determined using a Wilcoxon Rank test, are indicated; **p < 0·01, ***p < 0·001, ****p < 0·0001. Note that ten patients in total were treated in the trial [25], but one patient had samples taken at different times, preventing their inclusion in this analysis. (c) Loss of CD56^bright^ NK cells from the circulation at the peak of NK cell activation. The percentage of CD56^bright^ NK cells (defined as CD56^bright^, CD16^neg/low^, CD3^neg^) was determined as a fraction of the total NK cell population during reovirus treatment; this panel shows one representative patient. (d) Percentage of CD56^bright^ NK cells as a fraction of the total NK cell population in nine reovirus treated patients. Statistically significant changes in the size of the CD56^bright^ NK cell population were determined using a Wilcoxon Rank test; *p < 0·05

Amongst the genes downregulated in NK cells following reovirus treatment of PBMC was the sphingosine‐1‐phosphate receptor, S1PR1 (~5‐fold reduction; Figure 2b), a regulator of lymphocyte trafficking [55]. For mouse B and T lymphocytes, IFN‐I‐induced CD69 expression decreases sphingosine‐1‐phosphate receptor activity, contributing to the retention of these cells in lymph nodes [56]. We analysed changes in S1PR1 gene expression alongside CCR7, a chemokine receptor implicated in lymph node homing [57]. Reovirus treatment was associated with significantly reduced S1PR1 and increased CCR7 mRNA (Figure 6a). Coupled with the IFN‐I‐induced expression of CD69 both in vitro (Figure 1a and Figure S3) and in vivo [25], this phenotype suggested that reovirus treatment might alter the tissue distribution of NK cells during therapy.

Using blood samples from reovirus‐treated patients, we previously showed that the peak of NK cell CD69 expression coincided with the peak IFN‐I response [25]. We analysed CD69 expression in these patients in more detail, gating separately on CD56^bright^ and CD56^dim^ NK cell populations; CD69 expression was induced on both subsets with similar kinetics. Importantly, CD69 expression on both CD56^bright^ and CD56^dim^ NK cells was significantly enhanced from 1 to 48 h post‐treatment and significantly decreased from 48 to 96 h post‐treatment (Figure 6b), with peak CD69 expression in both subsets matching the peak IFN‐I response shown previously [25]. We analysed the relative abundance of the two NK cell subsets over the treatment course and found that, as a proportion of the total NK cells, the CD56^bright^ NK cells were significantly reduced in the blood 48 h post‐treatment (Figure 6c,d). Thus, for the CD56^bright^ NK cell subset, the expression of cell surface CD69 is inversely related to the abundance of these cells in the blood and, as CD69 expression dropped 96 h post‐treatment, the CD56^bright^ NK cells showed significantly increased abundance in the blood (Figure 6c,d). These results reveal that oncolytic reovirus treatment transiently alters the tissue distribution of human NK cell subsets in vivo.

## DISCUSSION

We have analysed the mechanisms by which oncolytic reovirus modulates human NK cell activity. A previous clinical trial of intravenous reovirus in cancer patients identified a peak of NK cell activation approximately 48 h post‐infusion. This was co‐incident with the maximal IFN‐I response as determined by ISG expression in leucocytes. Not surprisingly, NK cell activation in these patients bore the hallmarks of an IFN‐I response [25]. To the best of our knowledge, detailed studies of human NK cell activation by OV have not been previously undertaken. However, human NK cell activation by OV is well documented, with reovirus (dsRNA), Coxsackie virus (+ssRNA) and HSV‐1/T‐Vec (dsDNA) all showing IFN‐I dependent NK cell activation [24, 25, 30, 31, 32]. However, the source of OV‐induced IFN‐I can vary, with reovirus and T‐VEC both revealing an important role for monocyte‐derived IFN‐I but Coxsackie virus demonstrating the importance of plasmacytoid DC in IFN‐I‐dependent NK cell activation [24, 30, 32]. This may stem from the differential expression of particular pattern receptors (such as nucleic acid sensors) by different cell types and the nature of the corresponding OV genome and might also incur differential responses in the NK cells.

The kinetics of NK cell activation in oncolytic reovirus‐treated cancer patients resemble those found in both mice and humans [2, 26, 58, 59, 60, 61]. Our results demonstrate that reovirus‐induced IFN‐I acts directly on NK cells, inducing STAT1 phosphorylation and a characteristic transcriptional profile. The ISGs expressed by NK cells following reovirus treatment include many that interfere with various stages of viral entry, replication and egress [62, 63]. In addition, analysis of the interferome database coupled with prior studies confirms that components of the NK cell cytotoxic machinery are induced by IFN‐I [42, 64, 65, 66]. These results show that NK cells respond to IFN‐I in two ways; like other cells they increase their own antiviral defence mechanisms and, in addition, they increase expression of cytotoxic components to eliminate infected cells. This allows NK cells to destroy infected cells whilst minimizing their own infection. Such activity is likely to be important in the action of OV as it allows NK cells to kill tumour cells whilst protecting themselves against possible OV infection.

In mouse models, IFN‐I induces IL‐15 production by DC which then activates NK cells [42, 43] and for human NK cells, enhancement of reovirus‐mediated NK cell cytotoxicity is IL‐15 dependent [67]. Reovirus treatment induced several genes encoding cell cycle functions in NK cells and analysis using the interferome database suggested that they are not direct targets of IFN‐I, but are more likely induced by other cytokines, with IL‐15 being a prime candidate [54, 68]. This is consistent with data from mouse models showing that IL‐15 blockade following IFN‐I treatment inhibits NK cell proliferation, but not cytotoxicity [42]. Although reovirus treatment‐induced numerous cell cycle genes and their protein products, we did not detect the induction of NK cell proliferation, even when prolonging our assays for up to five days post‐reovirus treatment. We considered the possibility that reovirus and IFN‐I were priming NK cells for subsequent proliferation (e.g. by inducing the expression of IL15 and IL15RA genes), or that the weak STAT5 phosphorylation we detected in CD56^bright^ NK cells was simply reflective of low levels of IL‐15 in these assays. However, blocking and reconstitution experiments showed that reovirus treatment blocks IL‐15‐mediated NK cell proliferation in an IFN‐I dependent manner. The pro‐cytotoxic, but anti‐proliferative action of interferon on lymphocytes is long established [69, 70] and, more recently, mouse NK cells lacking a functional IFN‐I receptor (*Ifnar* ^−/−^) were shown to exhibit enhanced proliferation following MCMV infection [71]. This IFN‐I mediated inhibition of proliferation is paradoxical given that NK cell expansion occurs after viral infection in mice and humans [71, 72, 73, 74, 75, 76]. However, meaningful comparisons of the kinetics of IFN‐I responses and NK cell proliferation in these diverse systems are difficult to make. It seems likely that NK cell proliferation occurs once the peak of the IFN‐I response has subsided; in the reovirus clinical trial, NK cell expansions were only detectable after the peak of NK cell activation and ISG expression [25]. Interestingly, for T cells, IFN‐I can have both pro‐ and anti‐proliferative functions dependent upon whether it signals before or after TCR signalling. This dichotomous activity is regulated by the balance of the pro‐proliferative STAT4 versus anti‐proliferative STAT1 in the pre‐ or post‐TCR stimulated cell [77, 78]. Furthermore, in some tumour cells, IFN‐I signalling results in the induction of the cyclin‐dependent kinase (CDK) inhibitor, p21^WAF1^ [79, 80]; such activity preceding TCR signalling (or in NK cells, IL‐15 receptor engagement) might prevent proliferation. We did not detect significant induction of CDKN1A (p21^WAF1^), CDKN1B (p27^KIP1^) or STAT4 mRNA in NK cells from reovirus treated PBMC, but STAT1 mRNA was highly induced. Furthermore, PI3K‐AKT signalling is essential for mouse NK cell proliferation [81] and IL‐15‐induced AKT phosphorylation was reduced in NK cells pre‐treated with reovirus; reduced AKT phosphorylation may also antagonize nuclear exclusion of p27^KIP1^, favouring inhibition of proliferation [82, 83].

Gene expression changes suggestive of an altered NK cell trafficking phenotype prompted us to analyse NK cell subset distribution in reovirus treated patients. These patients showed the selective loss of the CD56^bright^ NK cell subset from the blood 48 h post‐infusion. This timepoint coincides with the peak IFN‐I response following reovirus delivery *in vivo* [25]. The egress of NK cells from lymph nodes in response to S1P differs from B and T lymphocytes, with S1PR5 as well as S1PR1 regulating activity [55, 56, 84, 85]. However, only S1PR1 mRNA met our statistical criteria for differential expression following reovirus treatment. The CD56^bright^ NK cell subset is highly enriched in SLT and it appears likely that reovirus treatment reduces S1P‐mediated egress from SLT in an IFN‐I dependent manner. Reovirus treatment‐induced IFNG expression in CD56^bright^ NK cells and, by analogy with mouse models, the transient retention of IFN‐γ expressing CD56^bright^ NK cells in the SLT will favour Th1 differentiation and more effective cytotoxic T cell responses [86]. The induction of this phenotype by a single dose of reovirus likely contributes to the immunotherapeutic action of reovirus and other OV. However, S1P gradients also regulate NK cell trafficking to inflamed tissues [85] and, since we were only able to sample blood from these patients, we cannot rule out the possibility that the CD56^bright^ NK cells lost from the circulation have relocated to the tumour in the liver, where we previously showed reovirus to be replicating [36].

The anti‐tumour action of OV stems from the combination of direct tumour lysis and the induction of anti‐tumour immunity and is dependent upon NK cell activity [13, 18, 19, 20, 21]. Our results show that OV treatment regulates both NK cell cytotoxicity and the immunomodulatory activity of CD56^bright^ NK cells, in particular via IFN‐γ production and possible homing/retention in lymph nodes. In our clinical trial, we found that NK cell activation (as determined by CD69 expression) occurred after the first dose of reovirus, but that subsequent infusions did not result in NK cell activation. Our results now show that NK cell responses to reovirus include the induction of IFN‐I pathway antagonists which might underpin this refractory phenotype. Despite this, the initial NK cell response to reovirus/IFN‐I is sufficient to induce the transient loss of CD56^bright^ NK cells from the periphery. Our data suggest that a single dose of IV‐reovirus is sufficient to modulate NK cell activity *in vivo*. However, a more robust anti‐tumour response may result from multiple infusions which elicit additional effects, such as sustained direct tumour killing and the release of tumour associated antigens.

## AUTHOR CONTRIBUTIONS

Conceived and designed study: MW, AAM, LFW, GPC; performed experimental work: MW, LFW, SLP, EBW; performed bioinformatics analysis: MW, APD, GPC; designed, implemented and analysed clinical trial: AAM, MC, YES, TDH, GPC; wrote paper: MW and GPC with input from all authors. Pelareorep for the clinical trial and laboratory studies was provided by Oncolytics Biotech. Matt Coffey is an employee of Oncolytics Biotech with shares and stock options. Alan Melcher has previously received research grant funding from Oncolytics Biotech.

## Supporting information

Supplementary Material

Table S3

Table S4

Table S5
