# Supplementary material for: Oncolytic virus treatment differentially affects the CD56dim and CD56bright NK cell subsets in vivo and regulates a spectrum of human NK cell activity
Source: Immunology. 2022 Mar 9;166(1):104–20. doi: 10.1111/imm.13453 (PMC10357483; doi:10.1111/imm.13453)
Supplement: Supplementary file 1 — Supplementary Material [file IMM-166-104-s004.docx]

**Supplement**

Oncolytic virus treatment differentially affects the CD56^dim^ and CD56^bright^ NK cell subsets *in vivo* and regulates a spectrum of human NK cell activity.

Michelle Wantoch, Erica B. Wilson, Alastair P. Droop, Sarah L. Phillips, Matt Coffey, Yasser M. El-Sherbiny, Tim D. Holmes, Alan A. Melcher, Laura F. Wetherill and Graham P. Cook

**Contents:**

1. Supplementary Table S1
2. Supplementary Table S2
3. Legend to Supplementary Table S3 (Table S3 Excel file available on journal website)
4. Legend to Supplementary Table S4 (Table S4 Excel file available on journal website)
5. Legend to Supplementary Table S5 (Table S4 Excel file available on journal website)
6. Supplementary Figure Legends S1-5
7. Supplementary Figures S1-5.
8. **Supplementary Table S1: Antibodies used in this study:**

| **Immunoblotting** |  |  |  |  |
| --- | --- | --- | --- | --- |
| **Antigen** | **Species** | **Clone/ID** |  | **Manufacturer** |
| STAT1 | mouse | 1 |  | BD Biosciences |
| pSTAT1 (pY701) | mouse | 4a |  | BD Biosciences |
| STAT4 | mouse | Clone 8 |  | BD Biosciences |
| pSTAT4 (pY693) | mouse | 38 |  | BD Biosciences |
| STAT5 | rabbit | 3H7 |  | Cell Signalling Technology |
| pSTAT5 (Y694) | rabbit | C11C5 |  | Cell Signalling Technology |
| MCM4 | rabbit | Ab4459 |  | Abcam |
| Granzyme B | mouse | 2C5/F5 |  | BD Biosciences |
| β-Actin | mouse | AC-15 |  | Sigma |
|  |  |  |  |  |
| **Flow cytometry** |  |  |  |  |
| **Antigen** | **Conjugate** | **Clone/ID** | **Isotype** | **Manufacturer** |
| CD56 | PE-Vio770 | REA196 | Human IgG1 | Miltenyi |
| CD56 | APC | AF12-7H3 | IgG1 | Miltenyi |
| CD3 | FITC | UCHT1 | IgG1 | BD Biosciences |
| CD3 | BV421 | UCHT1 | IgG1 | BD Biosciences |
| CD69 | BV421 | FN50 | IgG1 | BioLegend |
| CD69 | FITC | FN50 | IgG1 | BioLegend |
| CD69 | PE | FN50 | IgG1 | BioLegend |
| CD317 (Tetherin) | PE | REA202 | Human IgG1 | Miltenyi |
| pY701 STAT1 | APC | REA345 | Human IgG1 | Miltenyi |
| pY693 STAT4 | PE | 38/p-Stat4 | IgG2b | BD Biosciences |
| pY694 STAT5 | PerCP-Cy5.5 | 47/Stat5(pY694) | IgG1 | BD Biosciences |
| Granzyme B | PE | GB11 | IgG1 | Thermo Fisher Scientific |
| CD253 (TRAIL) | APC | RIK-2.1 | IgG1 | Miltenyi |
| CD16 | BV421 | 3G8 | IgG1 | BioLegend |
| CD16 | BUV395 | 3G8 | IgG1 | BD Biosciences |
| PCNA | PE | PC10 | IgG2a | BD Biosciences |
| Ki67 | PE | REA183 | Human IgG1 | Miltenyi |
| pY694 STAT5 | BV421 | 47/Stat5(pY694) | IgG1 | BD Biosciences |
| pS2448 mTOR | PE | O21-404 | IgG1 | BD Biosciences |
| pS473 AKT | Alexa Fluor 488 | M89-61 | IgG1 | BD Biosciences |
| CD107a | PE | H4A3 | IgG1 | BD Biosciences |
| **Isotype Controls** |  |  |  |  |
|  | BV421 | MOPC-21 | IgG1 | BioLegend |
|  | FITC | MOPC-21 | IgG1 | BD Biosciences |
|  | PE | REA293 | Human IgG1 | Miltenyi |
|  | APC | REA293 | Human IgG1 | Miltenyi |
|  | PE | 27-35 | IgG2b | BD Biosciences |
|  | PerCP-Cy5.5 | MOPC-21 | IgG1 | BD Biosciences |
|  | PE | MOPC-31C | IgG1 | BD Biosciences |
|  | VioBlue | REA293 | Human IgG1 | Miltenyi |
|  | PE | MOPC-173 | IgG2a | BD Biosciences |
|  | BV421 | X40 | IgG1 | BD Biosciences |
|  | Alexa Fluor 488 | MOPC-21 | IgG1 | BD Biosciences |

1. **Supplementary Table S2: qRT-PCR primers**

**2.1 SYBR Green method**

CDK2 F: ATGGATGCCTCTGCTCTCACTG R: CCCGATGAGAATGGCAGAAAGC

CCNA2 F: CTCTACACAGTCACGGGACAAAG R: CTGTGGTGCTTTGAGGTAGGTC

CCNB1 F: GACCTGTGTCAGGCTTTCTCTG R: GGTATTTTGGTCTGACTGCTTGC

**2.2. Taqman method**

ABL1: Applied Biosystems Hs01104728_m1

CCR7: Applied Biosystems Hs01013469_m1

S1PR1: Applied Biosystems Hs01922614_s1

MCM4: Applied Biosystems Hs00907398_m1

IFNG: Applied Biosystems Hs00989291_m1

1. **Supplementary Table S3**

Table S3 Excel file available on journal website.

**Legend:** Gene expression profiling of NK cells following reovirus treatment. The table shows the 1742 differentially expressed genes (FDR<0.05, fold change >1.5X) that were either upregulated (939 genes; highlighted in green) or downregulated (803 genes; highlighted in red) in NK cells from reovirus treated PBMC compared to untreated PBMC. Data from this table were used to construct Figures 2a-e of the main manuscript.

1. **Supplementary Table S4.**

Table S4 Excel file available on journal website.

**Legend:** Pathways represented by differentially expressed genes identified using gene set enrichment analysis. The 1742 genes (listed in Supplementary Table S3) were inputted into the Enrichr tool (<https://amp.pharm.mssm.edu/Enrichr/>) and enriched pathways identified using the Reactome 2016 database. The table shows the output from Enrichr, listing the significantly enriched pathways (adjusted p<0.05), and those genes from the DEGs that are assigned to these pathways. Data from this table was used to construct Figure 2c of the main manuscript.

1. **Supplementary Table S5.**

Table S4 Excel file available on journal website.

**Legend:** Transcription factors predicted to be associated with the differentially expressed genes identified using gene set enrichment analysis. The 1742 genes (listed in Supplementary Table S3) were inputted into the Enrichr tool (<https://amp.pharm.mssm.edu/Enrichr/>) and candidate transcription factors analysed from the Encode and ChEA Consensus TFs from ChIP-X database. The table shows the output from Enrichr, listing the significantly enriched transcription factors (adjusted p<0.05) and those genes from the DEGs that are associated with these transcription factors. Data from this table was used to construct Figure 2c of the main manuscript.

1. **Supplementary Figure Legends.**

**Supplementary Figure S1**.

**Gating strategies to separate CD56^dim^ and CD56^bright^ NK cells**

**A)** Representative gating of CD56^dim^ and CD56^bright^ NK cells for Phos-Flow, surface and intracellular staining experiments.

**B)** Gating of CD56^dim^CD16+ and CD56^bright^ CD16- NK cells after 5 day CFSE culture experiments.

**C)** Gating used in cell sorting of CD56^dim^CD16+ and CD56^bright^ CD16- NK, after magnetic isolation of NK cells from PBMC following 48 hours of culture with or without reovirus.

**Supplementary Figure S2**.

**STAT phosphorylation dynamics in cytokine treated CD56^dim^ and CD56^bright^ NK cells.**

**A)** STAT phosphorylation in purified, total human NK cells. Cells were treated with 100 IU/mL IFN-I, 10 ng/mL IL-12 or increasing concentrations of IL-15 and IFN-I for 1 hour, and blotted for phosphorylated or total STAT1, STAT4 and STAT5 as indicated.

**B)** STAT phosphorylation in CD56^bright^ and CD56^dim^ NK cells (detected by intracellular staining and flow cytometry) in PBMC treated with 100 IU/ml IFN-α, 10 ng/ml IL-12 or 50 IU/ml IL-15 for 1 hour. The flow cytometry gating strategy is shown in Supplementary Figure 1A. Graphs show median fluorescence intensities (MFI), with mean and standard deviation from three donors, from CD56^dim^ (black bars) and CD56^bright^ NK cells (grey bars) as indicated. Data was analysed by one-way repeated measures ANOVA, followed by Dunnet’s multiple comparison test; *p<0.05, **p<0.01.

**C)** Intracellular staining and flow cytometry to detect STAT phosphorylation**.** PBMC were treated with 100 IU/ml IFNα, 10 ng/ml IL-12 or 50 IU/ml IL-15 for 1 hour. NK cells within the PBMC (detected using cell surface staining as shown in Supplementary Figure 1A) were evaluated for levels of phosphorylated STAT1, STAT4 and STAT5 by intracellular flow cytometry. Histograms show isotype control or phosphorylated protein staining in total NK cells (according to the key), representative of 3 donors.

**D)** Time course of STAT phosphorylation in CD56^bright^ and CD56^dim^ NK cells in PBMC treated with cytokines as in panel B, fixed at 1, 8 and 24 hours and analysed by intracellular staining and flow cytometry. Graphs show mean MFI and standard deviation from two donors. The colour key indicates cytokine treatment.

**E)** IFN-α production by PBMC following treatment with 1 MOI reovirus (or left untreated). Supernatants was collected and analysed by ELISA. IFN levels were not detectable in the untreated cells as shown by the arrows.

**Supplementary Figure S3**

**Reovirus-mediated activation of NK cells for gene expression profiling**

**A)** PBMC from five donors were treated with reovirus for 48hrs *in vitro* and the NK cells purified by magnetic immunoselection. Activation of the NK cells was verified by analysis of CD69 expression in all five donors. Donors 1, 2, 4 and 5 are shown in the left panels and donor 3 in the right panels. These NK cells were used as a source of mRNA for gene expression profiling.

**B)** Expression of CD69 on CD56^dim^ and CD56^bright^ NK cells. The values indicate the percentage of cells expressing CD69 (or not) for both CD56^bright^ NK cells (top two quadrants) and CD56^dim^ NK cells (bottom two quadrants) in both untreated PBMC and PBMC treated with reovirus for 48 hrs

**Supplementary Figure S4**

**Reovirus and IFN-I regulate NK cell cytotoxicity.**

**A)** Induction of TRAIL following reovirus treatment. The histogram shows TRAIL expression from a single representative donor, with NK cells from untreated, IL-15 or reovirus-treated PBMC along with the isotype control stain as indicated. The graph shows the MFI of TRAIL expression from three separate donors. Data were analysed by a repeated measures one-way ANOVA, with Tukey’s multiple comparison test; **p< 0.01; ns, not significant.

**B)** PBMC were cultured without stimulation (untreated) or with reovirus (MOI 1) for 48 hours, followed by co-culture with K562 target cells. Degranulation of NK cells determined by CD107a expression on CD56+ NK cells. Plots are representative of data obtained from 2 donors.

**C)** Granzyme B expression by CD56^dim^ and CD56^bright^ NK cells following reovirus treatment. The histograms show flow cytometry from a single representative donor, with the different treatments (and isotype control antibody) indicated, along with the NK cell subset analysed via gating. The graph shows the median fluorescence intensity (MFI) of granzyme B expression from three separate donors; data were analysed by a repeated measures one-way ANOVA, with Tukey’s multiple comparison test; **p< 0.01.

**D)** IFN-I mediated induction of granzyme B. Purified NK cells were left untreated or stimulated with 10ng/ml IL-15 or increasing amounts of IFN-I (50 IU, 100 IU and 200 IU) for 48 hrs. Granzyme B expression was determined by immunoblotting, using actin as a control.

**E)** Purified NK cells were left untreated (Un) or stimulated with 10ng/ml IL-15 or 100 IU of IFN-I for 48 hrs and used in a degranulation assay (as in panel B) against K562 cells. The experiment was performed in triplicate and analysed using a Students T test.

**Supplementary Figure S5**

**Ki67 protein expression is not upregulated with reovirus treatment.**

PBMC were cultured with (Reo) or without (Un) 1 MOI reovirus, or with 10 ng/ml IL-15, for 48 hours. Protein expression of Ki67 was analysed in CD56^dim^ and CD56^bright^ NK cell populations by intracellular flow cytometry.

**A)** Representative histograms of ki67 expression from 1 of 3 donors. Untreated cells were used to set a gate for ki67 low cells (98%) or ki67 high expressing cells (2%). This gate was used to determine ki67 expression in the Reo and IL-15 treated samples in both CD56^dim^ and CD56^bright^ NK cell subsets.

**B)** Data from three donors, showing the percentage of ki67 high expressing NK cells in untreated and reovirus treated PBMC. Differences between mean percentage values were analysed using a paired T test.

1. **Supplementary Figures S1-5**
